# Epidemiology of HPV Genotypes among HIV Positive Women in Kenya: A Systematic Review and Meta-Analysis

**DOI:** 10.1371/journal.pone.0163965

**Published:** 2016-10-20

**Authors:** Sonia Menon, Aibibula Wusiman, Marie Claude Boily, Mbabazi Kariisa, Hillary Mabeya, Stanley Luchters, Frode Forland, Rodolfo Rossi, Steven Callens, Davy vanden Broeck

**Affiliations:** 1 International Centre for Reproductive Health (ICRH), Ghent University, Ghent, Belgium; 2 McGill University, Department of Epidemiology and Biostatistics, Montreal, Canada; 3 Faculty of Medicine, School of Public Health, Imperial College, London, United Kingdom; 4 CDC Foundation, Atlanta, GA, United States of America; 5 Moi University/Gynocare Fistula Centre, El Doret, Kenya; 6 School of Public Health and Preventive Medicine, Monash University, Victoria, Australia; 7 Centre for International Health, and Principal for Sexual and Reproductive Health, Burnet Institute, Victoria, Australia; 8 Royal Tropical Institute of the Netherlands/CEBHA, Amsterdam, the Netherlands; 9 Norwegian Institute of Public Health, Department of International Public Health, Oslo, Norway; 10 LSHTM Alumni, Geneva, Switzerland; 11 Department of Internal Medicine & Infectious Diseases, University Hospital, Ghent, Belgium; 12 AMBIOR, Laboratory for Cell Biology & Histology, University of Antwerp, Antwerp, Belgium; 13 National Reference Centre for HPV, Brussels, Belgium; Laboratory of Molecular Pathology, AML, Antwerp, Belgium; University of Washington, UNITED STATES

## Abstract

**Background:**

There is a scarcity of data on the distribution of human papillomavirus (HPV) genotypes in the HIV positive population and in invasive cervical cancer (ICC) in Kenya. This may be different from genotypes found in abnormal cytology. Yet, with the advent of preventive HPV vaccines that target HPV 16 and 18, and the nonavalent vaccine targeting 90% of all ICC cases, such HPV genotype distribution data are indispensable for predicting the impact of vaccination and HPV screening on prevention. Even with a successful vaccination program, vaccinated women will still require screening to detect those who will develop ICC from other High risk (HR) HPV genotypes not prevented by current vaccines. The aim of this review is to report on the prevalence of pHR/HR HPV types and multiple pHR/HR HPV genotypes in Kenya among HIV positive women with normal, abnormal cytology and ICC.

**Methods:**

PUBMED, EMBASE, SCOPUS, and PROQUEST were searched for articles on HPV infection up to August 2^nd^ 2016. Search terms were HIV, HPV, Cervical Cancer, Incidence or Prevalence, and Kenya.

**Results:**

The 13 studies included yielded a total of 2116 HIV-infected women, of which 89 had ICC. The overall prevalence of pHR/HR HPV genotypes among HIV-infected women was 64% (95%CI: 50%-77%). There was a borderline significant difference in the prevalence of pHR/HR HPV genotypes between Female Sex workers (FSW) compared to non-FSW in women with both normal and abnormal cytology. Multiple pHR/HR HPV genotypes were highly prominent in both normal cytology/HSIL and ICC. The most prevalent HR HPV genotypes in women with abnormal cytology were HPV 16 with 26%, (95%CI: 23.0%-30.0%) followed by HPV 35 and 52, with 21% (95%CI: 18%-25%) and 18% (95%CI: 15%-21%), respectively. In women with ICC, the most prevalent HPV genotypes were HPV 16 (37%; 95%CI: 28%-47%) and HPV 18 (24%; 95%CI: 16%-33%).

**Conclusion:**

HPV 16/18 gains prominence as the severity of cervical disease increases, with HPV 16/18 accounting for 61% (95%CI: 50.0%-70.0%) of all ICC cases. A secondary prevention program will be necessary as this population harbors multiple pHR/HR HPV co-infections, which may not be covered by current vaccines. A triage based on FSW as an indicator may be warranted.

## Background

Human papillomavirus (HPV) is a sexually transmitted infection, and high-risk (HR) HPV DNA has been shown to be present in 99.7% of cervical cancers worldwide [1]. Over 200 HPV genotypes have been identified and are divided into high-risk (HR) and low-risk (LR) carcinogens depending on their potential to cause cancer. HR types include 16, 18, 31, 33, 35, 39, 45, 51, 52, 56, 58, 68 and 59. Others are classified as potential High risk (pHR) (types 53, 66, 70, 73, 82). HPV 16 and HPV 18 are the most virulent HR-HPV genotypes, causing about 70% of all invasive cervical cancer (ICC) in the world [2].

Apart from a higher prevalence and broader range of HR HPV, HIV immunosuppression has been linked to multiple HPV infections [1, 3–5]. The inability to clear HPV infections and the reactivation of latent HPV infections, a result of immune suppression, have been attributed to multiple HPV genotype co-infections [6, 7].

Moreover, HIV positive women are more at risk for progression to cervical intraepithelial neoplasia grade 3 (CIN3). In 2007, the World Health Organisation (WHO) included ICC to the stage “4” of the AIDS classification of its clinical staging and case definition of HIV for resource-constrained settings [8].

Several countries, including Kenya, have licensed and adopted the bivalent HPV vaccine (Cervarix^™^) that protects against HPV genotypes 16 and 18 and the quadrivalent vaccine (Gardasil^™^) that protects against HPV genotypes 6, 11, 16 and 18 [9]. However, HPV vaccine uptake has been limited, with a recent longitudinal study in Eldoret, Kenya, reporting that only 31% (79/254) of those who entered the follow-up study to have been vaccinated [10].

In 2014, a nonavalent vaccine, not yet commercialized in Kenya, containing additional HPV types 6, 11, 16, 18, 31, 33, 45, 52, and 58 antigens will have direct implications for cervical cancer incidence and prevention in all regions of the world with the potential to prevent almost 90% of ICC cases worldwide.

Whilst the immunogenicity of both the bivalent and quadrivalent vaccines has also been recently documented in HIV positive women, the effectiveness of these vaccines in curbing the incidence of ICC is contingent upon the prevalence of oncogenic vaccine genotypes in HIV positive women [11, 12].

Considering the very high incidence of HIV and ICC observed in sub-Saharan Africa, the HPV type distribution is still not well characterised among HIV-infected populations in the region [13]. Whilst in some European populations a positive association of HIV infection and ICC has been reported, the picture lacks clarity in Africa [14–16]. Additionally, with the establishment of HAART programmes and consequent increase in life expectancy in HIV-positive women, a change in the pattern of the burden of HIV-related cancers can be expected for these patients.

Preceded only by breast cancer, ICC is the second most prevalent cancer among women in Kenya, and its incidence is increasing [17]. Despite high ICC prevalence, cervical screening uptake is low, with a 2014 cross sectional study in the Kisumu East District of Nyanza Province reporting a 17.5% screening uptake [18]. A successful vaccination program will not obviate the need for women to undergo screening to detect other oncogenic HPV genotypes. The WHO now recommends that “once a woman has been screened negative, she should not be rescreened for at least 5 years, but should be rescreened within ten” [19]. However, there is current scant evidence to support the adequateness of this guideline in HIV-infected women.

Given the scarcity of resources, screening programs will need to be tailored to the human and financial resources of the region, and a triage for HPV screening among vulnerable women envisaged. In Kenya, as in many parts of sub-Saharan Africa, female sexual workers (FSW) bear the greatest burden of HIV infection, and as early as 1985, a study reported that HIV prevalence was as high as 61% among a group of FSW in Nairobi [20]. A recent study estimated that 5 percent of the urban female population of reproductive age could be sex workers, making this population particularly at risk for HIV-HPV co-infection. Some published studies have suggested differential prevalence of pHR/HR HPV infection among FSW, however there are no pooled estimates for this population [21–23].

The overarching aim of this review is to guide primary and secondary prevention of cervical cancer programs in Kenya and has as objectives: 1) determine the prevalence of pHR/HR HPV genotypes among HIV-infected women with normal cytology to ICC; 2) explore the differential prevalence of pHR/HR HPV infections among FSW and non FSW women; 3) establish the pooled estimates for different pHR/HR HPV genotypes in these populations; 4) determine the pHR/HR HPV genotypes and multiple HPV genotypes in Kenya among HIV positive women with normal cytology/abnormal cytology to ICC.

## Method

### Search Strategy and Selection Criteria

The search strategy was designed by a medical librarian to identify studies reporting HPV genotypes associated with normal, abnormal cytology and cervical cancer in HIV infected women living in Kenya. We conducted this systematic review and meta-analysis based on a pre-defined search protocol that conformed to the criteria set out by the Meta-Analysis of Observational Studies in Epidemiology (MOOSE) group and was in accordance with the PRISMA statement [24, 25]. (S1 PRISMA Checklist)

We systematically searched PubMed, EMBASE, PROQUEST, and SCOPUS without any language restrictions. Reference lists of all retrieved articles and previous systematic reviews were checked for further eligible publications up to August 2^nd^ 2016.

The domains of the search terms were HIV, HPV, cervical Cancer, incidence or prevalence, and Kenya. We combined HPV and cervical cancer with the Boolean operator “OR”, and the result was combined with the other terms with “AND”. Full search strategy for the databases is given in S1 Search Strategy.

Studies were eligible if they reported our main outcomes of interest, overall pHR/HR HPV frequency as well as specific pHR/HR HPV genotype frequency in both a general HIV study population or in HIV infected FSW study population. If the HPV prevalence was not disaggregated into pHR/HR HPV genotypes, or no frequency of pHR/HR HPV genotype, multiple pHR/HR HPV genotypes or/and no disaggregated frequency of pHR/HR HPV genotype provided for Kenya, the authors of the retrieved articles were contacted. Another inclusion criterion was that HPV testing had to be performed through validated and commercial or WHO CE labelled PCR testing. Studies were included if their design was a cohort, cross-sectional, case-control or case-case study. Potential duplicate data were searched for.

**Fig 1:**
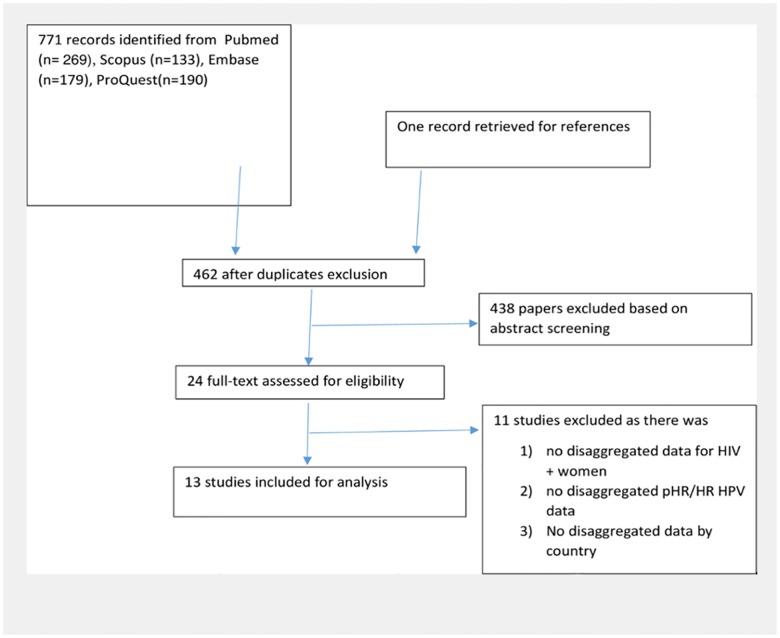
Flow diagram.

We excluded studies conducted on males, studies that did not provide pHR/HR HPV infection data by HIV status or if it was not possible to calculate the above information from data given (Fig 1).

For the first research question, data were extracted on the total number of HIV infected people with any pHR/HR HPV genotype. The prevalence among HIV infected women was calculated using the total number of women with pHR/HR HPV genotypes divided by the total number of HIV-infected women. We did not disaggregate by cytological status as we were interested in obtaining a general picture of the burden among HIV-infected women in Kenya, which would include women with ICC.

For the second research question, the pooled prevalence of pHR/HR HPV genotypes was compared between FSW and non FSW. As no cases of ICC have been observed among FSWs and ICC cases having a very high pHR/HR HPV genotype, the denominator included only HIV infected women with normal and abnormal cytology.

For the third research question, due to the small number of studies and study population, the frequency of pHR/HR HPV genotypes were only broken down into three groups: normal, abnormal cytology and ICC. Indeterminate cytological results were excluded, and inflammation was categorized as normal cytology.

For the fourth research question, the number of multiple infection pHR/HR HPV genotypes in HIV-infected women with normal to abnormal cytology was compared to the number of multiple infections in women with ICC. Their respective population was used as a denominator.

### Subgroup analyses

A sub-analysis was performed to assess differential prevalences during the total time of sample collection 1994–2008, by categorizing observations as pre-2004 or 2004–2008. This dichotomization was chosen to reflect changes in PCR sensitivities, changes of screening protocols, and the 2003 review of the epidemiological classification of oncogenic HPV genotypes [2].

### Data Abstraction

All studies were independently reviewed and critically evaluated for inclusion by two authors (SM and WA). All data was extracted independently and in duplicate manner by two investigators (SM and WA). The following items were recorded: first author, study period, publication year, study type, type of sampling, sample collection method, study population, total sample size, HIV and pHR/HR HPV prevalence of infected women, mean and standard deviation /median age with IQR number of women with specific pHR/HR HPV types if available, normal/abnormal cytology, invasive cervical cancer (ICC), HPV detection method, and data on FSW.

### Statistical Analysis

The HPV prevalence, type specific prevalence, 95% CI of prevalence of HPV infection, and specific HPV genotype were calculated according to Wald method. We used the DerSimonian and Laird random-effects model to pool overall HPV prevalence, as we expected the level of heterogeneity to be significant. The Q test was used to assess the presence of heterogeneity and the I^2^ index to quantify the extent of heterogeneity; p<0.10 was considered indicative of significant heterogeneity. To assess the robustness of pHR/HR HPV prevalence in HIV infected women, we performed a leave-one-out sensitivity analysis by iteratively removing one study at a time while recalculating the co-infection prevalence rate. Forest plots were produced to show the pooled estimate of the distribution of genotypes. Analysis was undertaken using STATA version 13 (Corporation, College Station, TX, USA). The STATA command Metaprop was used as it provides appropriate methods for dealing with proportions close to 0 [26].

### Ethical approval

No ethical approval was required as this was a meta-analysis (analysis of secondary data).

## Results

### Search results and study characteristics

**Fig 2:**
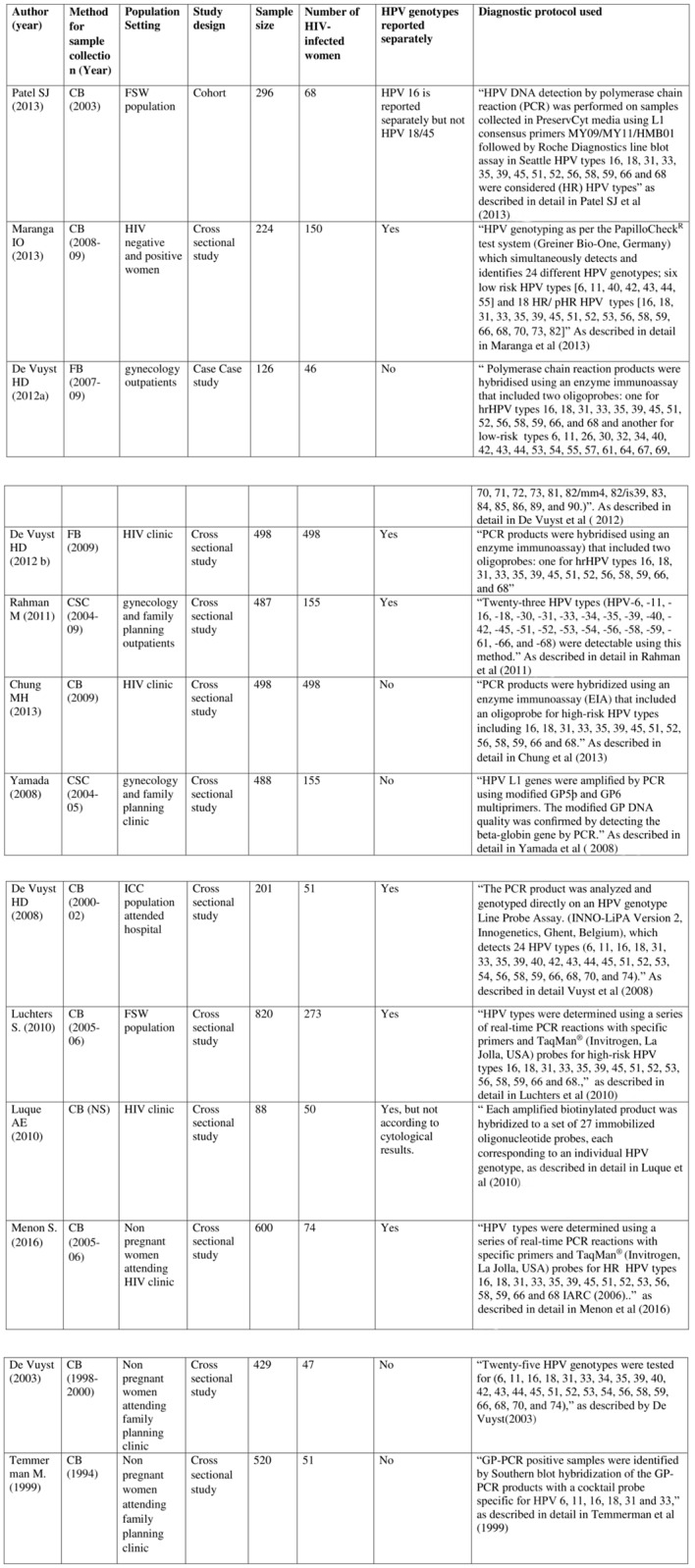
Studies included in the meta-analysis CB: Cervical brush; FB: Frozen biopsy; CSC: Cervical Scraped cell.

On August 2^nd^, 2016, we retrieved 771 studies from PUBMED, EMBASE, SCOPUS, and PROQUEST of which 310 were duplicates. We title/abstract-screened 461 articles, of which 29 studies were eligible for full text screening. Finally, 13 studies were included for this review (Fig 2). For pooling the pHR/HR HPV prevalence, all 13 studies reported this prevalence and hence were eligible. For pooling the different pHR/HR HPV genotypes, only 10 studies provided data on different HPV genotypes. Of these 10 studies, one could not be used due to lack of disaggregated pHR/HR HPV genotype data. Another study did not break down the HPV genotypes according to cytological results, and another study did not provide geographically disaggregated data, and so were also excluded from our analysis.

From the 7 eligible studies, 5 studies reported on pHR/HR HPV genotypes in HIV infected women with normal cytology and 6 reported on abnormal cytology. Three studies reported on frequency of pHR/HR HPV genotypes in HIV infected women with ICC.

From the 13 eligible studies, data on multiple pHR/HR HPV genotypes were only reported in 7 studies. Data were broken into two groups, normal/abnormal cytology versus ICC to juxtapose the difference in multiple pHR/HR HPV genotypes prevalence.

The 13 studies included in this meta-analysis yielded a total number of 2116 HIV positive women with individual sample sizes ranging from 46 to 498 (median = 74 women). Eleven studies were based on clinic-based recruitment in Kenya, (Rahman et al 2011 [27], Yamada et al 2008 [28], Vuyst et al 2003 [29] & 2012a [30] & 2012b [31], Temmerman et al 1999 [32] Vuyst et al 2008 [33] and Menon et al 2016) [34]; (Chung et al 2013 [35], Maranga et al 2013 [36], Luque et al 2010 [37]); one study used peer leader recruitment (Patel et al 2010) [38], and another used snowball sampling of FSW (Luchters et al 2010) [39]. Different protocols and techniques were used to determine HPV infection throughout Kenya. (Fig 2).

### Meta-analysis results

**Fig 3:**
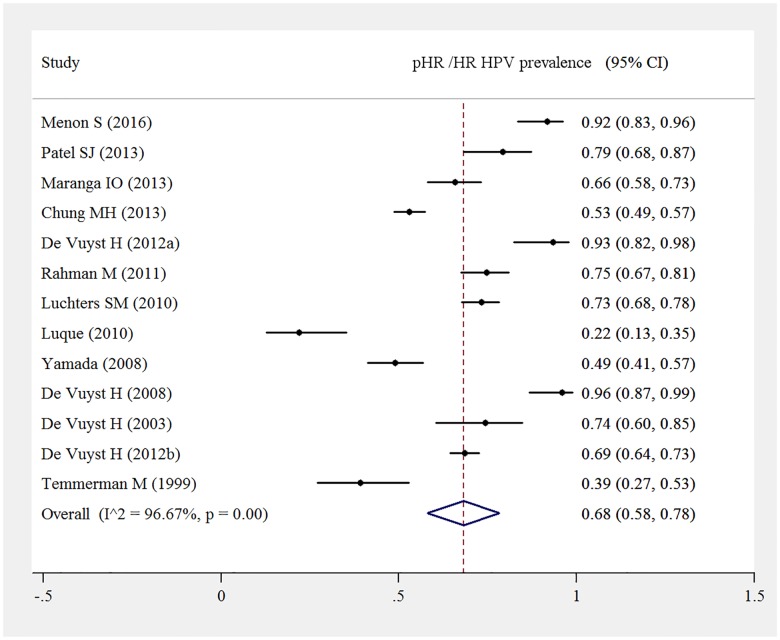
Pooled pHR /HR HPV prevalence among HIV-positive women.

Among the total 2116 HIV positive women, 1244 women were diagnosed with pHR/HR HPV infections. Pooled prevalence of pHR/ HR HPV genotypes among HIV infected women was 64% (95%CI: 50%-77%) and displayed significant heterogeneity, I^2^ = 98.1%, p<0.000. Pooled HPV prevalence was not substantially sensitive to the exclusion of any single study test (Fig 3).

**Fig 4:**
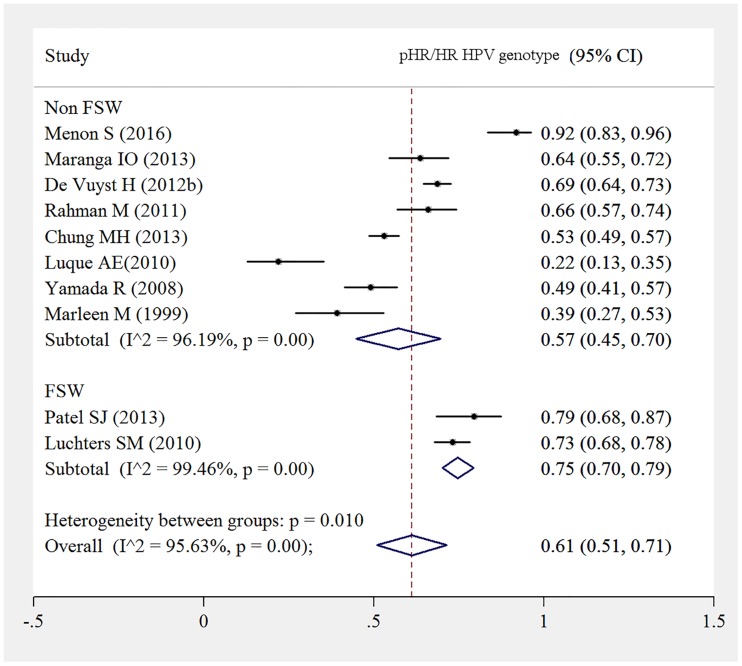
pHR/HR HPV prevalence in FSW versus non FSW.

There was a borderline statistically significant pooled HR HPV prevalence among FSW. Pooled HR HPV prevalence among FSW was 75% (95%CI: 70%-79%) compared to 57% (95%CI: 45%-70%) among non FSWs (Fig 4).

**Fig 5:**
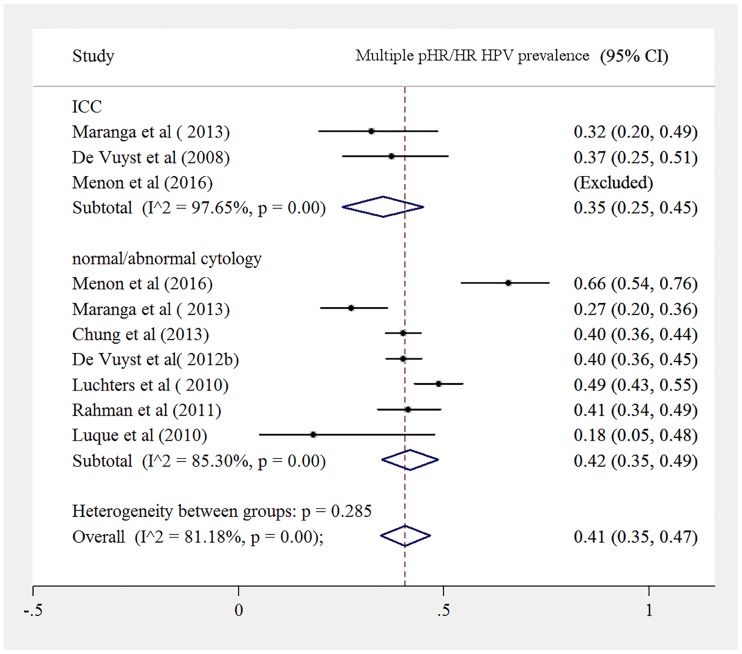
Prevalence of multiple pHR/HR HPV infection among HIV positive women, separated for women with normal cytology to HSIL and for women with ICC.

Both HIV infected women with normal cytology to HSIL and ICC had a high prevalence of multiple pHR HPV genotypes, 42% (95%CI: 35%; 49%) versus 35% (95%CI: 25%; 45%) (Fig 5).

**Fig 6:**
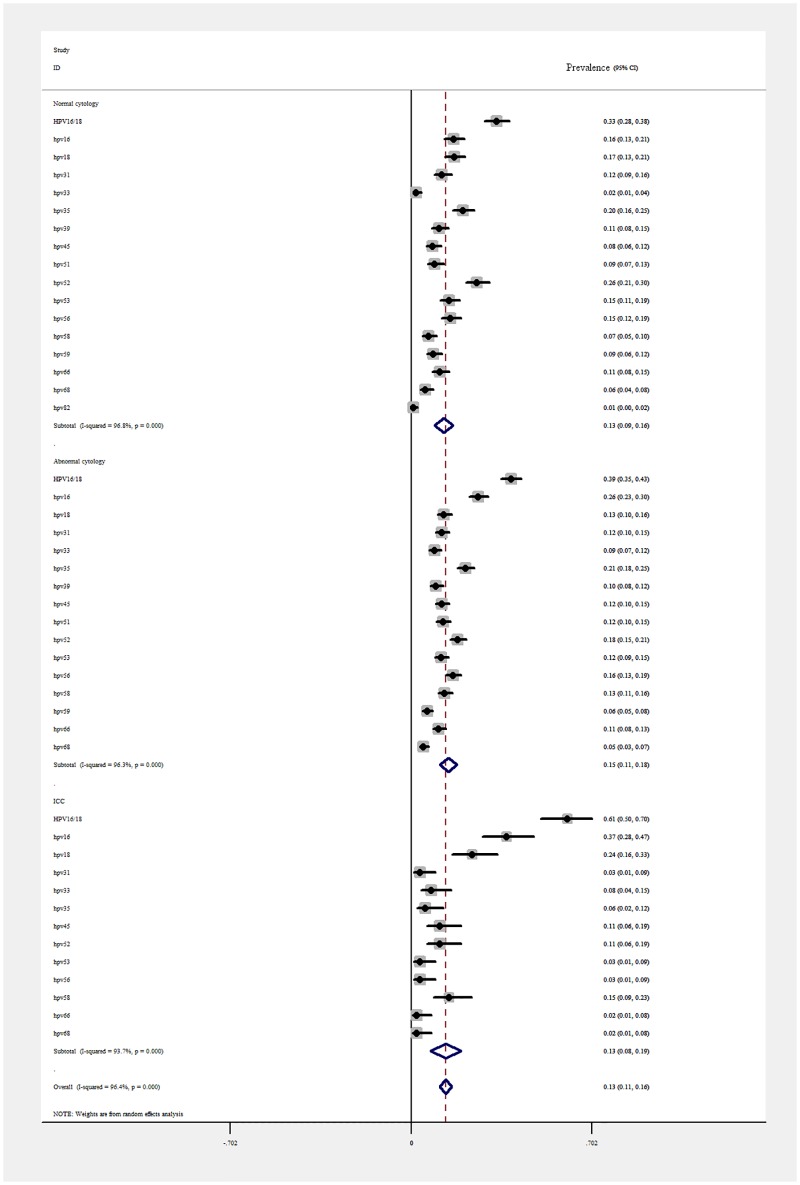
Pooled estimates of genotype specific pHR/HR HPV prevalence, separated for women with normal/abnormal cytology and women with ICC.

Out of the total number of HIV-positive women, the most prevalent HR HPV genotypes in women with normal cytology were HPV 52 with a pooled estimate of 26% (95%CI: 21%-30%-38.4; n = 92), followed by HPV 35, 20% (95%CI: 16% - 25%; n = 72). The most prevalent pHR/HR HPV genotypes in women with abnormal cytology were HPV 16 with 26%, (95%CI: 23%-30%; n = 156), followed by HPV 35 and 52, with 21% (95%CI: 18%-25%; n = 126) and 18% (95%CI: 15%-21%; n = 108), respectively (Fig 6).

In HIV infected women with ICC, the most prevalent HR HPV genotype in women were HPV 16 (pooled prevalence 37%; 95%CI: 28% - 47%; n = 33), followed by HPV18 (24%; 95%CI: 16%-33%; n = 21), followed by a 15% prevalence of HPV 58 (95%CI: 9%-23%; n = 11). HPV 31, 33, 35, 53 and 56 were also detected (Fig 6).

**Fig 7:**
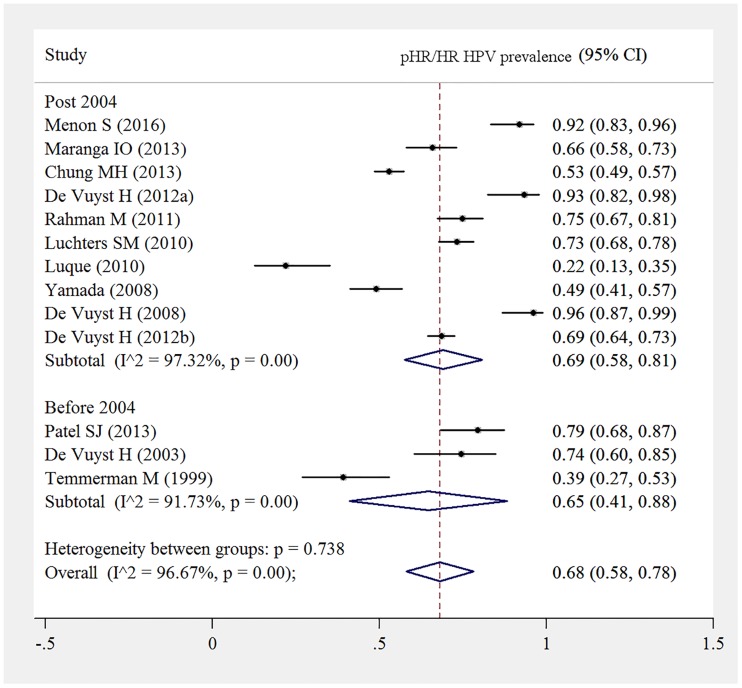
pHR/HR HPV prevalence among HIV-positive women stratified by pre 2004 and post 2004 time period. This forest plot reports the prevalence of pHR/HR HPV in HIV-infected women with normal cytology to ICC according to pre 2004 to after 2004.

In our subgroup analysis, time period did not appear to impact the pHR/HR HPV prevalence, with pooled estimates overlapping, pre 2004 65% (95%CI: 44%-88%) and post 2004, 64% (95%CI: 50%-77%) (Fig 7).

## Discussion

Our meta-analysis demonstrated a high burden of HR-HPV genotypes in a general HIV population, significantly higher than the recent meta-analysis on pooled HPV prevalence in the general female population in eastern Africa (42.2%) [40].

### Prevalence of specific PHR/ HR HPV genotypes in HIV-infected women with abnormal cytology

It was also found that, whilst HPV 52, followed by HPV 35, 53 and 56 were the most prevalent pHR/HR HPV genotypes detected in HIV positive women with normal cytology, in women with abnormal cytology, HPV 16 shifted to first place and was followed by HPV 35 and 52. Nevertheless, in women with ICC, HPV 18 gained prominence, ranking second after HPV 16.

Data on HPV type distribution in invasive and pre-invasive cervical cancer is required to predict the future impact of HPV16/18 vaccines, the future nonavalent vaccine and HPV-based screening test. Our systematic review suggests that HPV 16 regains it prominence in HIV women with abnormal cytology, though this difference was not statistically significant.

Our high pooled estimates suggest a prominent place for non 16/18 HPV types, including HPV 35, 52, 56, 58, 53, and 31 in HIV infected women with abnormal cytology. Similar observations have also been made from previous studies carried out in Africa and in the world where detection of non-16/18 HPV type patterns in HIV infected individuals is commonplace [41] [42].

Our pooled estimates are also in line with those of a 2015 meta-analysis on the general female population with LSIL from sub Saharan Africa for which HPV 16, 35, 52, 18, 53, 56, 58, 51, 45 and 66 were the top ten most common genotypes. Albeit, we do not have pooled estimates for HSIL, the ranking of HPV 16, followed by HPV 35 and 52 observed in our study is in line with that observed in eastern and southern regions of Africa where HPV 16 was estimated at 28%, followed by HPV 52 (16.5%) and HPV 35 (15.1%).

The relatively high pooled prevalence of HPV 53 in our meta-analysis suggests that HPV 53 plays a more prominent role in HIV infected women. Our pooled estimate in abnormal cytology of 12% is about twice as high as the pooled estimate found in Eastern African women (LSIL: 7.1% and HSIL: 5.9%), as well as the pooled 6.8% estimate in HIV infected women with LSIL and the 2.1% estimate observed in women with HSIL by Clifford et al (2006) [43].

### Prevalence of specific PHR/ HR HPV genotypes in HIV-infected women with ICC

The preponderance of both HPV 16 and HPV 18 in ICC cases in our meta-analysis is in agreement with the findings of the systematic review undertaken in Uganda and with the recent meta-analysis of HPV prevalence in women with normal cervical cytology to neoplasia in Africa [3, 40]. However, our pooled HPV 16/18 estimate of 61% (95%CI: 50.0%-70.0%) is lower than the 75.4% observed in Eastern African women with ICC.

Compared to the pooled estimates of 4.1% reported in the meta-analysis of HPV prevalence in eastern African women with ICC, we found a HPV 52 prevalence of 11% (95%CI 6.0%-19.0%) which was also present in single-type infections, However, our study found a similar prevalence of HPV 45, 11% (95%CI:6.0%-19.0%) compared to 9.1%.

In this review, we found that the prevalence of multiple HR HPV co-infections was analogous in women with normal cytology/HSIL to that of women with ICC, which may suggest that a synergistic interaction between pHR/HR HPV genotypes remains high in HIV women.

In our analysis comparing FSW who are at higher risk for STIs, our meta-analysis suggested that the prevalence of having pHR/HR HPV genotypes in FSW was statistically significantly higher than in non FSW. However, our subgroup analysis of pre-2004 compared to post 2004, revealed no statistically significant differences in pooled pHR/HR HPV prevalence.

### Strengths and limitations

To our knowledge, this systematic review represents the first attempt to evaluate the prevalence of HPV infections in HIV infected women, prevalence of different HR HPV genotypes in HIV infected women with normal, abnormal cytology and ICC in Kenya, and the second one on the sub Saharan continent attempting to measure the burden of HPV infection and ICC [44]. Another strength of this study is that it includes studies from different settings, including a family planning clinic, HIV clinics, and a community-based setting, which enabled us to capture a more representative HIV positive female population in Kenya. In addition to the diversity of settings and median age of the study population, the catchment of clinics may have differed in terms of socioeconomic status, which as a corollary, may have had an impact on the levels of immunosuppression.

The data should be interpreted in light of a number of limitations, including the small number of studies examining genotypes specifically in HIV infected women, especially with ICC as an endpoint. Our sub analysis did not reveal any statistically significant differences between pre 2004 and post 2004 suggesting that heterogeneity may not have significantly caused by an increase of PCR sensitivity, a wider range of pHR/ HR HPV genotypes testing over time, and a review of classification of HPV genotypes. However, of consideration is that some of the tests described are performed at analytical sensitivity while others have been validated clinically and use a clinical threshold, rendering a high variability of PCR outcome.

Another cause of heterogeneity may be that HIV-infected women with low CD4 counts are at risk for reactivation of HPV. However, a meta regression for age, CD4 cell count, HIV viral load or antiretroviral treatment could not be performed, as no individual information was available for these clinical parameters, which would allow adjustment or stratification of type-specific HPV prevalence for these variables. The high heterogeneity may be attributed to the different settings from which the study population was derived, including family planning facilities, HIV clinics and community-based settings. The high I2 that we encountered is in line with that observed in the 2015 meta-analysis from sub-Saharan Africa, where heterogeneity was found to be substantial, ranging from ICC: I^2^ = 88.8% to 99.1% in women with normal cytology [40]

In addition, another limitation relates to the cross-sectional analysis of studies with cross sectional study designs as well as baseline data on HPV and HIV derived from cohort studies. The temporal criterion of causality is not fulfilled, therefore reversal causality cannot be excluded.

### Potential reduction of ICC following an effective vaccine

Given that very few studies looked at type distribution in ICC in HIV positive women in Kenya, it is difficult to estimate the potential effectiveness of the bivalent or nonavalent vaccines. The still poorly characterised association between HIV and ICC causes a public health concern in Kenya, which currently has 1.6 million HIV-positive women who may well have an increased risk of developing ICC [45].

Data from the present meta-analysis indicate that current HPV 16/18 genotypes can be found in 61% of women with ICC. However, pooled estimates suggest that a 90% reduction of cervical cancer afforded by the nonavalent vaccine may be attained.

Whether Gardasil and Cervarix can attain a 70% reduction of cervical cancer may be contingent upon the natural history of the imputed pHR/HPV genotype in cancer genesis. However, with the high presence of other pHR/HR HPV genotypes in HIV infected women with ICC in Kenya, it is currently not possible to attribute a direct association between a specific genotype in cancer genesis as its relevance may be inflated.

In order to assess the potential reduction of ICC cases by 70%, an important research gap to address is the vaccines’ cross protection against HPV 45. Studies have shown that both current vaccines Gardasil and Cervarix afford cross protection against type 45, with Cervarix offering a higher degree of effectiveness [46]. However, this cross protection still has to be determined in HIV infected women.

Apart from limited information on cross protection, it should be established whether any concurrent HPV types may augment or decrease the efficacy of HPV vaccines, as a result of competition among the non-vaccinated HPV types [47]. These caveats and the pooled prevalence of above 90% of all the genotypes incorporated in the nonavalent vaccine outline the benefits of the nonavalent vaccine within this population.

### Secondary prevention

Secondary prevention, using cancer prevention tools such as visual inspection with acetic acid and/or Lugol’s iodine, or detection of high risk HPV genotypes will remain indispensable for various reasons, including the necessity of early identification of patients infected with HR/HPV genotypes not covered by current vaccines, as well as for post bivalent, quadrivalent and nonavalent vaccine surveillance and for unvaccinated young and older women. It is noteworthy that the impact of HPV vaccination will not be observed for years, especially with respect to types associated with pre-invasive and invasive cancers, as those tend to occur in older women.

Previous studies have shown a lack of association between HPV 16 and immunosuppression [7]. Hence, despite immune reconstitution, a pooled estimate of 26%, of older HIV infected women with abnormal cytology infected with HPV 16 or 37% of women with ICC in Kenya may still need to be monitored regularly. Although most HPV infections may resolve without treatment, the immuno-epidemiology of some genotypes is still poorly characterized, especially in regards to resolution of infections and regression of non-HPV 16 infections.

Because of the potential to alter their behavior in the post vaccine area and so resulting in case type replacement, the relatively high prevalence of HPV 53 and other undiagnosed pHR/HR HPV genotypes not covered by the nonavalent vaccine underscores the need for it to be considered in a screening protocol. This is hampered by the lack of affordable nucleoacid amplification methods, such as Papillomacheck, which requires specific apparatus [48, 49].

## Conclusion

Our pooled estimates of HPV 16 and 18 of 61% in HIV-infected women with ICC suggest the need for a wider protection that the nonavalent vaccine would confer.

Whilst HPV 52 is most prominent in HIV-infected women with normal cytology, in women with abnormal cytology, HPV 16 regains its preponderance and is followed by HPV 35, only to be replaced by HPV 18 in women with ICC.

To assess the potential vaccine efficacy in Kenya, the synergistic interactions between the multiple genotypes harboured by HIV positive women with premalignant and malignant diseases in the post vaccine era need to be elucidated.

Borderline statistically significant differences between pooled estimates of HR HPV genotypes in FSW and non FSW suggest that cervical cancer prevention may warrant a triage based on this indicator. An effective secondary prevention programme in HAART era, will require that the immuno- epidemiology of specific HPV types in HIV-infected women be explored.

## Supporting Information

S1 PRISMA Checklist (DOCX)

S1 Search Strategy (DOCX)

## Abbreviations

HIV: human immunodeficiency virus
HPV: Human papillomavirus
LSIL: Low-grade squamous intraepithelial lesions
HSIL: High-grade squamous intraepithelial lesions
ICC: Invasive cervical cancer
WHO: World Health organization
