# Supplementary material for: Epidemiology of HPV Genotypes among HIV Positive Women in Kenya: A Systematic Review and Meta-Analysis
Source: PLoS One. 2016 Oct 20;11(10):e0163965. doi: 10.1371/journal.pone.0163965 (PMC5072621; doi:10.1371/journal.pone.0163965)
Supplement: S1 Search Strategy — (DOCX) [file pone.0163965.s002.docx]

**Literature Search on HIV/HPV/Cervical Cancer Incidence/Prevalence in Kenya – All Years**

| **Database** | **Date Searched** | **Results** | **Unique Results** | **Duplicates** |
| --- | --- | --- | --- | --- |
| PubMed | 8/2/2016 | 269 | 269 | 0 |
| Scopus | 8/2/2016 | 133 | 62 | 71 |
| Embase | 8/2/2016 | 179 | 54 | 125 |
| ProQuest | 8/2/2016 | 190 | 76 | 114 |
| **TOTAL** |  | 771 | 461 | 310 |
|  |  |  |  |  |
|  |  |  |  |  |

**Database: PubMed**

**Date Searched: 8/2/2016**

**Results: 269/269 unique**

**Search Strategy:**

Search ((("Uterine Cervical Neoplasms/epidemiology"[Mesh] and "kenya"[title] AND Humans[Mesh])) OR ("Uterine Cervical Neoplasms"[Mesh]AND ("incidence"[mesh] or "prevalence"[mesh]) AND "kenya"[title] AND "humans"[MeSH Terms]))) OR ((("Uterine Cervical Neoplasms"[Mesh] AND ("incidence"[mesh] or "prevalence"[mesh]) AND ("kenya"[mesh] OR "kenya"[title] AND "humans"[MeSH Terms]))) AND ("Uterine Cervical Neoplasms/epidemiology"[Mesh] and ("kenya"[mesh] OR"kenya"[title]) AND Humans[Mesh]))

OR

Search (((((("kenya"[mesh] AND ("Papillomaviridae"[Mesh]) AND ("Incidence"[Mesh] OR "Prevalence"[Mesh] OR "Epidemiology"[Mesh] OR "epidemiology" [Subheading])) AND Humans[Mesh])) OR ("kenya"[title] and "human papillomavirus"[title] AND Humans[Mesh])) OR (((("kenya"[title] and "human papillomavirus"[title] AND Humans[Mesh])) OR (("kenya"[mesh] AND ("Papillomaviridae"[Mesh]) AND ("Incidence"[Mesh] OR "Prevalence"[Mesh] OR "Epidemiology"[Mesh] OR "epidemiology" [Subheading]))] AND Humans[Mesh])) AND Humans[Mesh])) OR (((kenya*[title] or "nairobi"[title]) and "hpv"[title]) AND Humans[Mesh])) OR ((("incidence"[title] or "prevalence"[title]) and "kenya"[all fields] and ("hpv"[title] or "human papillomavirus"[title])) AND Humans[Mesh]) Filters: Humans

OR

Search (("HIV Infections/epidemiology"[majr] OR "HIV Infections/statistics and numerical data"[Majr] )) AND "Kenya"[Mesh] and ("incidence" or "prevalence")

**Database: Scopus**

**Date Searched: 8/2/2016**

**Results: 133/62 unique**

**Search Strategy:**

( ( TITLE ( kenya* OR nairobi ) AND TITLE-ABS-KEY ( "cervix neoplasms" OR "cervical cancer" ) AND TITLE ( epidemiology OR incidence OR prevalence ) OR ( ( TITLE-ABS-KEY ( kenya* OR nairobi ) AND TITLE ( "cervix neoplasms" OR "cervical cancer" ) AND TITLE ( epidemiology OR incidence OR prevalence ) ) OR ( ( TITLE ( kenya* OR nairobi ) AND TITLE ( "cervix neoplasms" OR "cervical cancer" ) AND TITLE-ABS-KEY ( epidemiology OR incidence OR prevalence ) )

( TITLE ( kenya* OR nairobi ) AND TITLE ( "cervix neoplasms" OR "cervical cancer" ) AND TITLE-ABS-KEY ( epidemiology OR incidence OR prevalence ) )

( TITLE-ABS-KEY ( kenya* OR nairobi ) AND TITLE ( "cervix neoplasms" OR "cervical cancer" ) AND TITLE ( epidemiology OR incidence OR prevalence ) ) ( TITLE ( kenya* OR nairobi ) AND TITLE-ABS-KEY ( "cervix neoplasms" OR "cervical cancer" ) AND TITLE ( epidemiology OR incidence OR prevalence ) )

( TITLE ( kenya* OR nairobi ) AND TITLE ( "cervix neoplasms" OR "cervical cancer" ) AND TITLE ( epidemiology OR incidence OR prevalence ) )

(TITLE(kenya* or nairobi) AND TITLE(hpv or "human papillomavirus")AND TITLE(epidemiology or incidence or prevalence))

(TITLE(kenya* or nairobi) AND TITLE(hiv or "human immunodeficiency virus")AND TITLE(epidemiology or incidence or prevalence))

**Database: Embase (1947 - August 1, 2016)**

**Date Searched: 8/2/2016**

**Results: 179/54 unique**

**Search Strategy:**

1

(Kenya and (hpv or human papillomavirus) and (incidence or prevalence)).mp.

2

(kenya and (hiv or human immunodeficiency virus) and (incidence or prevalence)).mp.

3

limit 2 to (english language)

4

(kenya and (hiv or human immunodeficiency virus) and (incidence or prevalence)).ti.

5

limit 4 to (english language)

6

cervical cancer.mp. or uterine cervix cancer/

7

Kenya*.ti. and (cervical cancer.ti. or uterine cervix cancer/) and (incidence or prevalence).ti.

8

limit 4 to (english language)

**Database: ProQuest**

**Date Searched: 8/2/2016**

**Results: 190/76 unique**

**Search Strategy:**

Limit to English

S10

1 or 4 or 5 or 6 or 7 or 8 or 9

S9

(all(Kenya*) AND all((incidence OR prevalence)) AND ti(("cervical cancer" OR "cervix neoplasms"))) OR (all(Kenya*) AND all((incidence OR prevalence)) AND all(("cervical cancer" OR "cervix neoplasms")))

S8

all(Kenya*) AND all((incidence OR prevalence)) AND all(("cervical cancer" OR "cervix neoplasms"))

S7

all(Kenya*) AND all((incide OR prevalence)) AND ti(("cervical cancer" OR "cervix neoplasms"))

S6

all(Kenya*) AND ti((incidence OR prevalence)) AND ti(("cervical cancer" OR "cervix neoplasms"))

S5

ti(Kenya*) AND ti((incidence OR prevalence)) AND ti(("cervical cancer" OR "cervix neoplasms"))

S4

ti(Kenya*) AND ti((incidence OR prevalence)) AND ti("cervical cancer" OR "cervix cancer" OR "cervix neoplasms")

S3

ti(Kenya*) AND ti((incidence OR prevalence)) AND ti(hiv OR "human immunodeficiency virus")

S2

all(Kenya*) AND all((incidence OR prevalence)) AND all(hiv OR "human immunodeficiency virus")

S1

all(hpv OR "human papillomavirus") AND
